# Adherence to quality of care measurements among 58,182 patients with new onset diabetes and its association with mortality

**DOI:** 10.1371/journal.pone.0208539

**Published:** 2018-12-12

**Authors:** Beatriz Hemo, Danit R. Shahar, Dikla Geva, Anthony D. Heymann

**Affiliations:** 1 Maccabi Healthcare Services, Tel Aviv, Israel; 2 The S. Daniel Abraham International Center for Health and Disease, Department of Public Health, Faculty of Health Sciences, Ben-Gurion University, Beer-Sheva, Israel; 3 The Department of Family Medicine, The Sackler Faculty of Medicine, University of Tel Aviv, Tel-Aviv, Israel; University of Adelaide School of Medicine, AUSTRALIA

## Abstract

**Objectives:**

Disease registry for diabetes care encourages transparency and benchmarking of quality of care (QoC) measurements for all service providers and seems to improve diabetes care. This study evaluate changes over time in QoC measurement performance in a large diabetes registry among newly diagnosed diabetics and it association with mortality.

**Methods:**

Retrospective cohort study of patients in a large health maintenance organization diabetes registry from years 2000 to 2013. We identified 58,182 patients diagnosed with diabetes from 2000–2008 and examined the level of performance for seven QoC measurements (HbA1c, LDL, albumin-creatinine-ratio, fundus/foot examinations, BMI and Blood-pressure) at diagnosis year. We also searched data regarding visits to dietitians or endocrinologists, and purchase of diabetes and statin medications. We used Mantel-Haenszel's **χ**^2^ test to assess QoC performance and mortality rate by calendar year of entry into the registry, and Cox regression to calculate hazard ratios (HRs) and 95% confidence intervals (CIs) for all-cause mortality up to 5 years from diagnosis adjusted for age, gender, socio-economic status and comorbidities.

**Results:**

The total QoC measurements improved from a mean of 2.71 tests performed in 2000 to 5.69 in 2008 (p<0.001). The mortality rate dropped from 7.7% in 2000 to 5.7% in 2008 (p<0.001). Patients with more QoC measurements performance who visited a dietitian and purchased statin medications had a lower mortality risk (HRs (95% CIs) 0.89 (0.87–0.92), 0.83 (0.76–0.91) and 0.70(0.65–0.75) respectively). Visits to endocrinologists and purchases of oral diabetes medication and insulin were associated with a higher risk of mortality (HRs (95% CIs) 1.20(1.07–1.35), 1.35(1.26–1.46) and 3.36(2.92–3.87) respectively).

**Conclusion:**

Performance of QoC measurements including visiting a dietitian and purchase of statin medications were associated with lower mortality in patients with diabetes. It may be that the early active involvement of the patients in their care plays a protective role in long term mortality.

## Introduction

The global prevalence of diabetes among adults 18 years and above has risen from 4.7% in 1980 to 8.5% in 2014, and diabetes is expected to become the seventh most common cause of death in the world [1]. The trend in Israel is similar, with a prevalence of diabetes among adults aged 18+ years in 2011 of 9.1% that increased to 9.7% in 2015 [2]. Patients with diabetes are at increased risk for morbidity and mortality compared to the general population [3–6].

Diabetes Registries aid health care providers in establishing standardized criteria for follow-up of patients, targeted interventions and calculation of quality measures. Quality of care measures can be calculated on the basis of automated patient care data recorded for registry members [7–8].

Israel established a national program for quality assessment of community healthcare in 2004 [9]. Each measure assesses the proportion of patients in a given target who received a specific service known to be associated with high-quality care. The program [10] has reported substantial improvements in most QoC measurements during the years 2008–2010. An ecological study demonstrated an association between improvement in performance for these quality indicators for diabetes and improvement in diabetes-related health status and mortality [11].

Numerous healthcare organizations have built diabetes registries in order to consistently identify their members with diabetes. Registries enable the collection of data from all medical interactions in order to generate feedback for the primary care teams. Performance on standard quality of care QoC measures reflects the extent to which providing feedback to care providers improves outcomes [7,12–13]. Process QoC measures generally focus on the proportion of patients in the target population who receive specific interventions, for example foot examinations, eye examinations and laboratory monitoring. A study of patients with DM2 in eight Western New York counties showed that patients treated in practices that maintained diabetes registries demonstrated better compliance with recommended laboratory tests and a lower hospital utilization rate than patients treated in other practices [14].

QoC measures are developed on the basis of data regarding factors associated with improved outcomes derived from observational studies and large-scale clinical trials. Each measure is an indicator of the extent to which a patient receives recommended monitoring and treatment. QoC measures, such as the monitoring of glycemic control, to assess outcome is well-recognized [15–16]. However data on the association of level of performance for QoC measures and the incidence of diabetic complications and mortality in real-life situations among new onset diabetics are scarce. It is important to measure the extent to which new onset diabetics receive optimal monitoring and treatment (as measured by QoC measures) during the first year after a new diabetes diagnosis, and whether delivery of optimal care at the start is in fact associated with improved outcomes in the long term.

The present study used data from a large diabetes registry to evaluate changes over time in performance of QoC measures among new onset type 2 diabetics. We then assessed the relationship between performance of QoC measurement and all-cause mortality rate in this population.

## Materials and methods

### Setting

We used the diabetes registry of Maccabi Healthcare Services (MHS) to conduct this retrospective cohort study. MHS is Israel's second largest HMO serving over 2 million members and covering 25% of the population nationwide. MHS maintains computerized databases of services provided to members, including physician visits, hospital admissions, laboratory tests and pharmacy purchases. Physician visit records include at least one diagnosis coded according to ICD-9. The Israeli health system provides universal coverage for a comprehensive basket of health services [17] with few dropouts, and it is therefore assumed that the vast majority of services received by MHS members are captured by the MHS administrative data system. Personal identity numbers allow linkage of all patient care data at the individual level, and also enable linkage to other MHS registries, such as the cardiovascular registry and the chronic kidney disease registry. Information on vital status is updated on a monthly basis by the National Insurance Institute. Socio-economic status (SES) was defined according to patient residence and the 2008 population census conducted by the Israel Central Bureau of Statistics. SES is ranked along a 10-point ordinal scale then classified into low (levels 1–4), medium (5–7), and high (8–10) groupings. Data are also obtained from the MHS cancer, renal disease and cardiovascular disease registries. Validation of these chronic disease registries has been described in detail elsewhere [18–19].

In 2002, the MHS developed a model that links performance measurement to a strategy for improving healthcare [20]. QoC measures for patients with diabetes were among the first to be included. As of 2004, the MHS was required to report similar QoC measurements annually to the Ministry of Health as a part of National Program for Quality Indicators [9].

### Study population in the diabetes registry

The MHS diabetes registry was established in 1999 and has been described in detail elsewhere [21–22]. Briefly, the registry includes patients who were diagnosed as having diabetes according to laboratory criteria of glucose or glycated hemoglobin (HbA1c) level and/or diabetic medication purchase. The criteria for entry into the diabetes registry are based on the disease criteria suggested by the American Diabetes Association (ADA) [23], including fasting blood glucose of ≥126 mg/ml or a casual plasma glucose concentration of ≥200 mg/dl. The registry is ongoing, updated and validated by physician feedback. There is incomplete differentiation in the registration between Type 1 and Type 2 diabetes. For this reason, consequently, we limited the study population to new onset diabetics age 40 and older at diagnosis such that the likelihood of including Type 1 diabetes was extremely small. Women diagnosed with gestational diabetes were also excluded from the current analysis.

The study population included members of the registry over the age of 40 years who first met registry entrance criteria between January 1, 2000 and December 31, 2008. The entry date to the registry served as the index date for the purpose of follow-up. The total follow-up period lasted until study closure on December 31, 2013, death or leaving MHS whichever occurred first.

### Measurements

The MHS has established several QoC measurements to assess treatment given to patients with diabetes [22]. The QoC considered in this study were 1) Glycosylated hemoglobin (HbA1c), 2) low density lipoprotein (LDL), 3) albumin/creatinine ratio (ACR), 4) measurement of weight and height for the calculation of body mass index (BMI), 5) Fundus exam, 6) foot exam, and 7) measurement of blood pressure. Compliance with a measure was defined as receipt of the service within one year of a diabetes diagnosis.

For each patient we calculated the total number of QoC tests performed (of a possible 7 in total) during each twelve-month period following the index date. In order to assess severity of disease at the time of diagnosis, we searched pharmacy purchasing records to determine whether there had been at least one purchase of medication from any of the following groups during the first year after the index date: oral diabetes medications (sulfonylureas, biguanides or any oral medication), insulin treatment, and lipid-lowering drugs (statins). In addition, we searched provider visit records for visits with endocrinologists and dietitians in the first year following index date. It is important to emphasize that the QoC measures considered here are process and not outcome measures and it was not our intention to use these measures to evaluate the level of control (for example of HbA1c). Therefore, all study subjects could be included in the analysis, whether or not specific measures were performed.

In order to identify patients diagnosed as having cancer, cardiovascular or renal disease prior to index date, we linked the study group to appropriate registries that record these patient details. A previous history of cancer was defined as the presence in the MHS cancer registry of diagnoses prior to the index date. A subject was deemed to have renal disease if estimated glomerular filtration rate (eGFR) was less than 60 (ml/min/1.73m^2^) on at least one occasion, or eGFR was greater than 60 (ml/min/1.73m^2^) with at least two abnormal urinary protein results during the three months prior to or following the recorded eGFR. Subjects appearing in the MHS cardiovascular disease registry with a diagnosis of congestive heart failure, atrial fibrillation, myocardial infarction, ischemic heart disease, cerebrovascular accident or transient ischemic attack prior to the index date were considered to have a diagnosis of cardiovascular disease. The cardiovascular diseases definitions have been described in detail elsewhere [18].

### Statistical analyses

We examined the distribution of demographic and clinical characteristics of the study population overall and by calendar year of entry into the registry. Changes in the distribution of these characteristics were tested for statistical significance with the Mantel-Haenszel's **χ**^2^ test for linear trend for categorical variables and Student’s t test or ANOVA for continuous variables. P < .05 was considered significant for all analyses. Cox proportional hazard models was used to estimate Hazard Ratios (HRs) for mortality with 95% confidence intervals (CIs) up to 5 years after the index date, adjusting for such factors as age at entry, gender, SES level and presence of comorbidities. Follow-up was limited to five years in order to allow for complete assessment of outcomes for all subjects, regardless of calendar year of entry. Our first model included only measures of services during the first year after diabetes diagnosis. To allow for the effect of a previous cancer diagnosis on survival, we stratified the Cox proportional hazard models by cancer history status. The second model included the total number of QoC tests performed in each year of follow-up as a time-dependent variable. The follow-up period was until one year prior to death, leaving MHS or the end of the study period, whichever occurred first. The third model was limited to study subjects who received HbA1c, LDL and blood pressure measurement within the first year following diagnosis, and included the mean values of these measures recorded during the index year, as well as the total number of QoC test performed as a time-dependent variable. In addition, we analyzed the association between years of follow-up and trends of change in those three indices results using generalized estimating equation (GEE) model with repeated measures over time. These models were adjusted for all variables in study: year of diabetes diagnosis, total performance of QoC tests during the first year of diagnosis, age, gender, SES level, visits to dietitians and endocrinologists, purchase of diabetes and statin medications and cardiovascular and renal disease prior to the diagnosis of diabetes. The analysis was carried out using SPSS version 22 (SPSS Inc., Chicago, IL) and STATA version 12 (StataCorp LLC, College Station, Texas).

Approval was obtained from the Institutional Review Board (IRB) and Ethics Committee (Helsinki) of MHS—Bayit Balev, Helsinki approval number 3/2014.

## Results

As of 2008, 84,876 MHS members had been included in the diabetes registry. Of these 58,182 met the criteria of new onset diabetes for the period of the study. Table 1 shows the number of new onset diabetics calendar year and the distribution of demographic and health characteristics in the index year. The number of newly diagnosed diabetics was quite stable over the years, i.e., about 6,000 patients per year.

**Table 1:** New onset diabetics by year of diagnosis and baseline characteristics.

	Diabetes diagnosis year								
	2000	2001	2002	2003	2004	2005	2006	2007	2008
**Total new onset diabetics**	**6,597**	**7,412**	**6,475**	**6,385**	**6,588**	**6,352**	**6,445**	**6,374**	**5,554**
**Demographic & health characteristics**									
mean age**	60.24	60.48	59.56	59.92	60.38	60.28	60.20	59.89	59.84
median age	59.78	59.89	58.16	58.02	58.46	58.70	58.92	58.92	58.98
Gender-male %	52.4	52.4	53.7	53.7	52.1	53.8	54.3	53.4	53.4
**QoC measurements**									
HbA1c test** **%**	**87.9**	**88.0**	**89.4**	**91.3**	**93.4**	**94.4**	**95.9**	**96.6**	**97.0**
HbA1c mean	7.76	7.45	7.35	7.33	7.23	7.03	7.13	7.15	7.26
SES: low	24.8	23.2	24.9	24.5	23.1	24.4	24.5	22.7	24.9
medium	58.3	59.6	57.4	57.9	58.3	58.0	57.8	59.1	58.2
High	17.0	17.2	17.7	17.7	18.6	17.6	17.8	18.2	16.8
ACR test** **%**	**18.6**	**24.7**	**29.8**	**37.5**	**43.9**	**58.9**	**71.4**	**78.3**	**80.1**
ACR mean**	47.50	36.84	26.87	28.27	26.65	27.96	28.42	26.22	29.60
BMI measurement** **%**	**0.5**	**6.7**	**15.6**	**19.4**	**31.7**	**48.6**	**68.4**	**83.1**	**88.5**
BMI mean**	32.55	31.61	31.68	31.99	31.44	31.56	31.41	31.29	31.49
LDL test** **%**	**95.7**	**96.8**	**97.2**	**98.5**	**98.8**	**99.2**	**99.3**	**99.5**	**99.2**
LDL mean**	124.90	125.08	127.66	135.74	134.73	133.34	128.76	124.88	122.19
**Blood pressure** **measurement %**	**76.6**	**77.4**	**76.1**	**72.6**	**73.3**	**74.4**	**77.2**	**79.7**	**81.1**
Systolic mean**	145.10	143.04	138.35	135.97	135.17	135.12	134.73	134.09	134.18
Diastolic mean**	83.26	82.65	81.88	81.49	80.92	80.94	80.62	80.28	80.14
Fundus examination** %	21.1	23.4	25.9	29.2	34.2	38.7	44.2	53.3	57.1
Foot examination** %	2.0	1.8	4.8	7.1	9.3	12.9	21.6	37.6	49.0
Total QoC measurements**, mean	2.71	2.89	3.38	3.78	4.09	4.51	5.00	5.47	5.69
Endocrinologist visits** %	12.1	10.8	11.3	12.5	11.3	14.1	12.8	15.5	17.6
Dietitian visits** %	1.8	18.4	39.8	43.2	43.5	45.8	47.9	47.9	48.1
**Drug purchases yes/no (**at least 1 purchase = 1) **%:**									
Sulfonylureas**	29.2	23.2	19.5	16.3	14.1	14.1	13.0	11.0	12.2
Biguanides**	36.5	35.3	38	41.2	40.0	44.6	47.9	51.9	59.7
Purchase at least 1 oral drug **	51.1	47.2	46.9	48.1	45.6	49.9	52.5	55.2	63.5
Insulin purchase**	3.9	2.8	2.5	2.6	2.8	3.6	3.6	4.0	6.2

Variables presented as mean ± SD or %

* P < .05 and

**P≤.001

Abbreviations: HbA1c, (hemoglobin A1c); BMI, body-mass index; ACR, albumin/creatinine ratio)mg/dl); LDL, low-density lipoprotein(mg/dl); SES, socio-economic status

**Fig 1:**
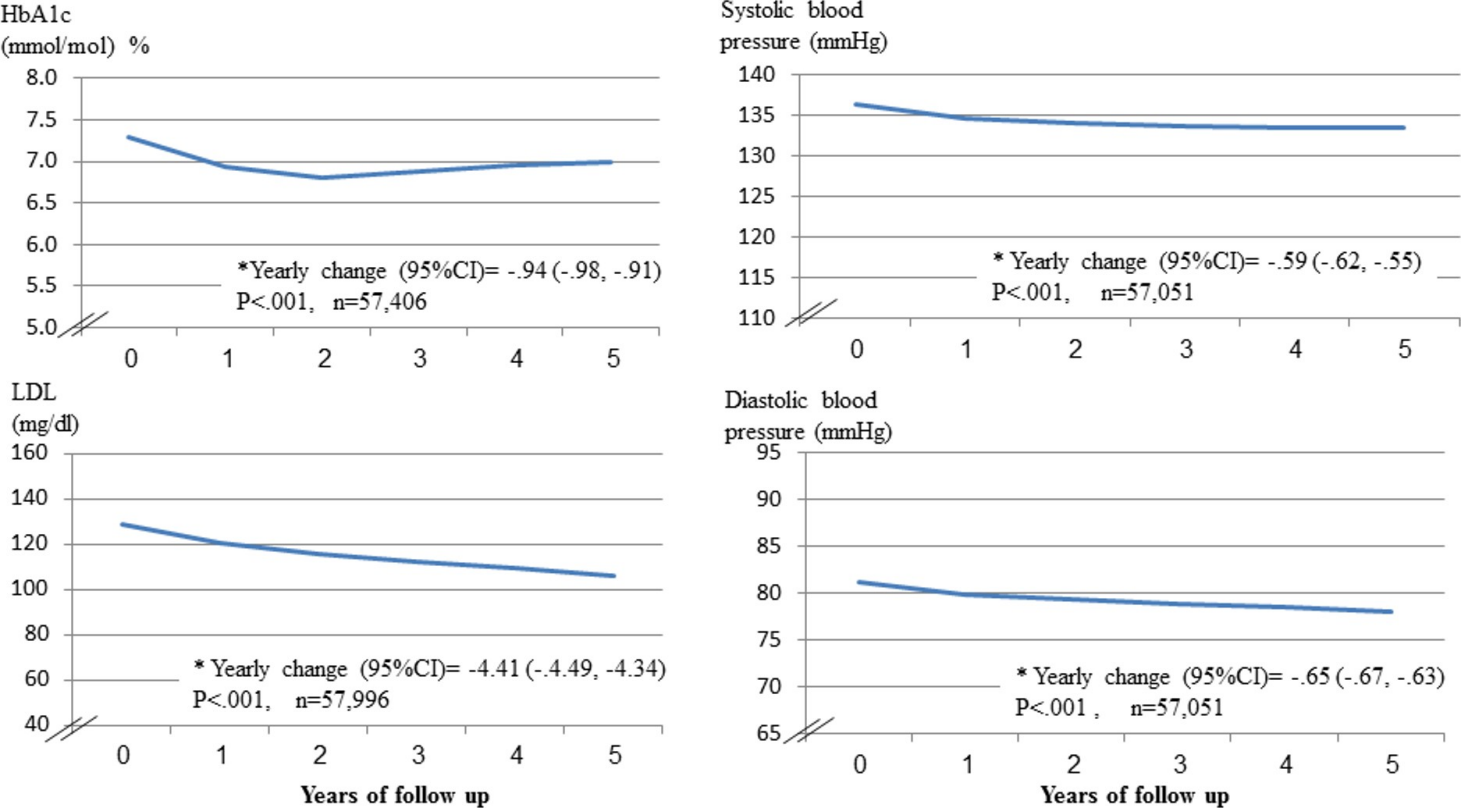
Trends in levels of Hba1c, LDL, systolic and diastolic blood pressure during five years of follow-up. Abbreviations: LDL **=** low density lipoprotein. ^*^ Analysis using Generalized Estimating Equation, the coefficients (95% confidence interval) indicate mean change in level of each QoC measurements values per year, adjusted for all variables in study: year of diabetes diagnosis, total performance of QoC tests during the first year of diagnosis, age, gender, socio-economic status, visits to dietitians and endocrinologists, purchase of diabetes and statin medications and cardiovascular and renal disease prior to the diagnosis of diabetes.

While mean age distribution varied significantly during the period of the study, these differences were not felt to be of clinical significance (Table 1). Gender and SES distribution did not varied significantly during the period of the study. With the exception of percent compliance with LDL testing, which was already high in 2000, the rate of performance of the other quality measures increased significantly over the period of the study. With regard to diabetes treatment, there was a reduction in the use of sulfonylureas drugs and an increase in the use of biguanides (from 29.2% to 12.2% [p<0.001] and from 36.5% to 59.7% [p<0.001], respectively) over time. Overall medication use in the first year of diagnosis increased with calendar year of diagnosis (from 51.1% to 63.5% [p<0.001]). Insulin use during the first year after diagnosis nearly doubled over this period, rising from 3.9% in 2000 to 6.3% in 2008 (P<0.001). We observed an increase in the performance of QoC measurements between 2000 and 2008. Mean number of QoC measures performed per patient rose from 2.71 to 5.69. Moreover, among patients who performed HbA1c or LDL tests or blood pressure measurement, we observed a trend of improvement over the years in the mean values of these indices (Fig 1).

Table 2 shows the mortality rate in the first year of diagnosis and from the second year onward. Calendar year of diagnosis was not associated with the risk of mortality within the first year of the index date (χ^2^_(MH)_ = 3.21, *p* = .073). There was, however, an apparent reduction in mortality from the second year of diabetes diagnosis onwards during the period of the study, from 7.7% among diabetics diagnosed in 2000 to 5.7% in diabetics diagnosed in 2008 (χ^2^_(MH)_ = 38.94, *p* < .001). The standardized mortality rate (SMR), adjusted for age, among new onset diabetics over years 2–5 of the study period shows an excess mortality among the diabetes patients compare to all MHS population age 40+ in every diagnosis year.

**Table 2:** Mortality among new onset diabetics by year of diagnosis.

Year of diagnosis	Newly diagnosed diabetics	Mortality in the first year after diagnosis		Remaining newly diagnosed diabetics	Mortality 2–5 years after diagnosis	
	N	N (%)	SMR (95%CI)	N	N (%)	SMR (95%CI)
2000	6,597	108 (1.6)	1.12 (0.90–1.34)	6,489	500 (7.7)	1.41 (1.29–1.53)
2001	7,412	115 (1.6)	1.06 (0.86–1.26)	7,297	512 (7.0)	1.24 (1.12–1.36)
2002	6,475	107 (1.7)	1.16 (0.94–1.38)	6,368	381 (6.0)	1.22 (1.10–1.34)
2003	6,385	119 (1.8)	1.28 (1.04–1.52)	6,266	417 (6.7)	1.32 (1.20–1.44)
2004	6,588	116 (1.8)	1.43 (1.18–1.88)	6,472	405 (6.3)	1.33 (1.19–1.47)
2005	6,352	140 (2.2)	1.61 (1.22–1.76)	6,212	411 (6.6)	1.49 (1.35–1.63)
2006	6,445	121 (1.9)	1.49 (1.22–1.76)	6,324	382 (6.0)	1.43 (1.29–1.57)
2007	6,374	109 (1.7)	1.43 (1.16–1.70)	6,265	360 (5.8)	1.45 (1.29–1.57)
2008	5,554	104 (1.8)	1.72 (1.39–2.05)	5,450	311 (5.7)	1.46 (1.30–1.62)
Total	58,182	1,039 (1.8)		57,143	3,679 (6.44)	
	χ^2^_(MH)_ = 3.21, df = 1, *p* = .073			χ^2^_(MH)_ = 38.94, df = 1, *p* < .001		

**SMR,** standardized mortality rate

Cox proportional hazard models were applied to estimate mortality Hazard Ratios (HRs) at 2–5 year after diabetes diagnosis (mean follow-up 4.88 years, median follow-up 5.0 years); all the variables met the criteria of proportionality over time (Table 3). This analysis excluded patients who died (n = 1,039) during the first year of diagnosis, assuming that most of those deaths were not due to complications of diabetes. A total of 57,143 newly diagnosed diabetics were included in the analysis, 3,679 of whom died within study years 2–5. The year of diabetes diagnosis and the patient’s age, gender, SES, history of cardiovascular and kidney disease were included in the model for adjustment.

**Table 3:** Cox proportional hazard models for prediction of mortality at 2–5 years following diagnosis with malignancy vs. patients with no history of malignancy.

	Model 1		Model 2	Model 3
	Prediction of death among patient with malignancy (N = 3,789, events = 689)	Prediction of death among patient with no history of malignancy (N = 53,354, events = 2,990)	Prediction of death among patient with no history of malignancy (N = 53,354, events = 2,990)	Prediction of death among patient with no history of malignancy (N = 35,827 events = 1,840)
Baseline characteristics at the first year of diagnosing:	HR(95% CI)	HR (95% CI)	HR (95% CI)	HR (95% CI)
Year of diabetes Diagnosis	1.05 (1.01–1.09)**	1.01 (0.99–1.02)	1.01 (1.00–1.03)	1.02 (0.99–1.04)
Gender (male = 1)	1.13 (0.97–1.32)	1.37 (1.28–1.48) ***	1.38 (1.29–1.50) ***	1.45 (1.32–1.60) ***
Age at diabetes diagnosis	1.04 (1.04–1.05) ***	1.10 (1.09–1.10) ***	1.09 (1.09–1.10) ***	1.09 (1.09–1.10) ***
SES: medium (reference low)	0.45 (0.38–0.54) ***	0.61 (0.57–0.66) ***	0.61 (0.57–0.66) ***	0.61 (0.57–0.68) ***
High (reference low)	0.44 (0.36–0.55) ***	0.48 (0.43–0.54) ***	0.48 (0.43–0.54) ***	0.48 (0.41–0.56) ***
History of cardiovascular disease (yes = 1)	1.49 (1.25–1.77) ***	1.64 (1.50–1.78) ***	1.67 (1.53–1.81) ***	1.77 (1.59–1.97) ***
History of kidney disease (yes = 1)	1.42 (1.17–1.72) ***	1.45 (1.30–1.62) ***	1.44 (1.29–1.60) ***	1.47 (1.29–1.68) ***
Visit endocrinology (yes = 1)	0.87 (0.67–1.12)	1.20 (1.07–1.35) **	1.21 (1.08–1.36) **	1.11 (0.97–1.28)
Visit dietitian (yes = 1)	0.91 (0.75–1.09)	0.83 (0.76–0.91) ***	0.83 (0.76–0.92) ***	0.88 (0.79–0.97) *
Purchase diabetes oral drug (yes = 1)	1.29 (1.11–1.50) **	1.35 (1.26–1.46) ***	1.34 (1.24–1.44) ***	1.18 (1.07–1.30) **
Purchase Insulin (yes = 1)	2.60 (1.88–3.60) ***	3.36 (2.92–3.87) ***	3.13 (2.72–3.60) ***	2.46 (2.05–2.97) ***
Purchase statin (yes = 1)	0.59 (0.50–0.69) ***	0.70 (0.65–0.75) ***	0.73 (0.68–0.79) ***	0.68 (0.62–0.75) ***
Total QoC measurements	0.89 (0.84–0.94) ***	0.89 (0.87–0.92) ***	0.83 (0.81–0.84) ***	0.86 (0.84–0.88) ***
Hba1c (mmol/mol) %				1.17 (1.13–1.21) ***
LDL (mg/dl)				1.00 (1.00–1.00)
Systolic blood pressure(mmHg)				1.00 (1.00–1.01) **
Diastolic blood pressure(mmHg)				0.99 (0.99–1.00)*

Model 1 includes baseline characteristics only. Model 2 include total QoC measurements as a time-dependent variable. Model 3 include total QoC tests as a time-dependent variable among patients with data extraction of Hba1c, LDL and blood pressure at time of diabetes diagnosis. Levels of significance are denoted as

* < 0.05

** < 0.01

*** < 0.001.

Abbreviations: LDL = low density lipoprotein; SES, socio-economic status

Table 3 presents the results of the Cox proportional hazards analyses. In model 1, among new onset diabetics with no history of cancer, variables that may indicate the severity of diabetes at the time of diagnosis, such as a visit to an endocrinologist, purchase of oral diabetic medication and purchase of insulin treatment were associated with mortality (HR 1.20 95% CI 1. 07–1.35, HR 1.35 95% CI 1.26–1.46, and HR 3.36 95% CI 2.92–3.87, respectively). A number of factors appear to be associated with reduced risk, among them a visit to a dietitian (HR 0.83 95% CI 0.76–0.91), total number of QoC measurements performed within one year of the index date (HR 0.89 95% CI 0.87–0.92), and purchasing of lipid-lowering treatment (statins) (HR 0.70 95% CI 0.65–0.75). For Model 2, we included QoC test performance as a time-dependent variable and found that compliance with QoC measurements over the course of the follow-up period was associated with reduced mortality (HR 0.83 95% CI 0.81–0.84). This effect remained after adjustment for level of Hba1c, LDL and blood pressure measures in the index year (Model 3—HR 0.86 95% CI 0.84–0.88).

## Discussion

In this retrospective cohort study of 58,182 newly diagnosed diabetes patients from the MHS diabetes registry we observed an improvement in the performance of QoC measurement tests over the years 2000–2008. While ACR and BMI were performed during the index year in less than 1.0% of diabetics diagnosed in 2000, the proportion rose to nearly 90.0% among diabetics diagnosed in 2008. Similarly, the proportion of subjects having a visit to a dietician rose from 1.8% among the 2000 cohort, to nearly 50.0% in the 2008 cohort. These findings are similar to findings from American Veterans Affairs system, which documented significant quality improvement in diabetes care indicators following reengineering at all levels of the healthcare system [24–25].

Structured QoC measures for monitoring the appropriate performance of diabetic care have become an established part of the MHS internal organizational assessment [20–21]. According to our findings, there was no decrease in the average age of the member at the time of diagnosis, but there did appear to be an increase in the intensity of treatment in the first year. Specifically, more patients received hypoglycemic oral medication therapy and insulin treatment.

There was a significant improvement in QoC measures, such as BMI, blood pressure, and foot and fundus examinations throughout the study period. This improvement in the recorded clinical measures may be partially due to better data recording, and partially to the investments MHS has made in organizational resources. Other health organizations with Healthcare Quality Registries, have also shown improved patient care [26–27]. However, we also observed QoC measurements reflecting the performance of laboratory tests and specialist consultations, over time, indicating that the observed improvement goes beyond enhanced documentation and truly reflects better provision of services. This reflects the MHS ongoing effort to use diabetes registry data to facilitate dynamic monitoring of selected indices of accepted patient care with the goal of improving outcomes.

Along with the improved performance for QoC measurements we documented a decrease in all-cause mortality for the five years following diagnosis from 7.7% in the 2000 diabetic cohort to 5.7% in the 2008 cohort. This finding corresponds with reports of a decrease in all-cause mortality among diabetics in Canada and the UK [28] during the period from1996 to 2009, and in a Danish population over a similar period as that of our study [29].

The quality of the care of the patient with diabetes in the first year of diagnosis, as measured by standard indicators, was associated with reduced risk for mortality. In particular, the total number of QoC measurements performed in the index year, at least one dietitian visit, and the purchase of statin medicine were associated with reduced mortality. Furthermore, numbers of QoC measurements performed in subsequent years were also associated with reduced mortality. Our finding that subjects who consulted an endocrinologist or purchased oral diabetic medication or insulin during the index year were at higher risk for mortality, suggest that these factors are indicators of increased disease severity at diagnosis.

Creating a diabetes registry with effective capture of patient care data facilitates standardized measurements of care quality. Our data indicate that improved performance for these measurements is correlated with improved outcomes. QoC measurements reflect a composite of the services provided by MHS the compliance of patients with recommended treatment and monitoring. It may be that the early involvement of patients in their care, as reflected in compliance with QoC measurements, plays a role in reducing mortality. One example of the effect is the strong observed association between visits to a dietician in the first year of diagnosis with reduced mortality; patients who were motivated to visit a dietitian may well have been more likely to meaningfully change their diet and lifestyle.

This study has several limitations and strengths. The main limitation was its retrospective nature based on administrative data. For example, we used a medication purchase as a proxy for the use of that medication. The data on SES was defined by area of residence and not by personal information. We focused on measures of performance rather than biometric or biochemical measurements. However, among the group of study subjects with consistent performance of laboratory examinations or blood pressure measurement, we observed trends of improvement over the course of the study period in mean values of HbA1c, LDL and blood pressure. Because the MHS provides a comprehensive basket of health services to all members, we assume that few patients seek care outside the system and that therefore utilization data used to calculate QoC measures are complete for all patients during the study period. This level of coverage, as well as the size of the covered population, represents important strengths of our study.

This study focused on the performance of QoC measures not on diabetes control. We explored the improvement in the performance of QoC measures over time and the association of performance with mortality. Patient compliance during the first year of diagnosis was associated with reduce mortality in the long term, and the association appears to be maintained among patients who remain compliant in subsequent years. Our findings support the policies of healthcare organizations that put emphasis on QoC measurements in the setting of diabetes care. It is not enough for each physician to treat his patient with diabetes to the best of his abilities; the healthcare organization must invest in disease management programs for their entire patient population based on QoC measurements.

## References

[1] Shaw JE, Sicree RA, Zimmet PZ. Global estimates of the prevalence of diabetes for 2010 and 2030. Diabetes Res Clin Pract 2010;87(1):4–14. 10.1016/j.diabres.2009.10.007 19896746 10.1016/j.diabres.2009.10.007

[2] The National Program for Quality Indicators for Community Health Care in Israel. Report for the years 2011–2015. Available from: http://healthindicators.org.il/wp-content/uploads/2014/05/English-report-2011-2015_final.pdf

[3] Barnett KN, Ogston SA, McMurdo ME, Morris AD, Evans J. A 12-yearfollow-up study of all-cause and cardiovascular mortality among 10,532 people newly diagnosed with type 2 diabetes in Tayside, Scotland. Diabet Med. 2010;27(10):1124–9. 10.1111/j.1464-5491.2010.03075.x 20854379 10.1111/j.1464-5491.2010.03075.x

[4] Smith NL, Barzilay JI, Kronmal R, Lumley T, Enquobahrie D, Psaty BM. New-onset diabetes and risk of all-cause and cardiovascular mortality. Diabetes Care. 2006;29(9):2012–7. 10.2337/dc06-0574 16936145 10.2337/dc06-0574

[5] Zucker I, Shohat T, Dankner R, Chodick G. New onset diabetes in adulthood is associated with a substantial risk for mortality at all ages: a population based historical cohort study with a decade-long follow-up. Cardiovasc Diabetol. 2017;16(1):105 10.1186/s12933-017-0583-x 28810857 10.1186/s12933-017-0583-x PMC5558697

[6] Tancredi M, Rosengren A, Svensson AM, Kosiborod M, Pivodic A, Gudbjörnsdottir S, et al Excess Mortality among Persons with type 2 Diabetes. N Engl J Med. 2015;373(18):1720–32. 10.1056/NEJMoa1504347 26510021 10.1056/NEJMoa1504347

[7] Arts DG, De Keizer NF, Scheffer GJ. Defining and improving data quality in medical registries: a literature review, case study, and generic framework. J Am Med Inform Assoc 2002; 9: 600–11. 10.1197/jamia.M1087 12386111 10.1197/jamia.M1087 PMC349377

[8] Gliklich R, Dreyer N, Leavy M, eds. Registries for Evaluating Patient Outcomes: A User’s Guide. Third edition Rockville, MD: Agency for Healthcare Research and Quality 4 2014 Available from: http://www.effectivehealthcare.ahrq.gov/registries-guide-3.cfm. 24945055

[9] Jaffe DH, Shmueli A, Ben-Yehuda A, Paltiel O, Calderon R, Cohen AD, et al Community healthcare in Israel: quality indicators 2007–2009. Isr J Health Policy Res. 2012;1:3 10.1186/2045-4015-1-3 22913466 10.1186/2045-4015-1-3 PMC3415131

[10] National Program for Quality Indicators in Community Healthcare in Israel 2008–2010. Available at: http://healthindicators.ekmd.huji.ac.il/reports/Israel%20quality%20indicators%202008–2010%20English.pdf.

[11] Calderon-Margalit R, Cohen-Dadi M, Opas D, Jaffe DH, Levine J, Ben-Yehuda A, et al Trends in the performance of quality indicators for diabetes care in the community and in diabetes-related health status: an Israeli ecological study. Isr J Health Policy Res. 2018 1 17;7(1). 10.1186/s13584-018-0206-3 PMC5773014 29343291

[12] Higashi T, Shekelle PG, Adams JL, Kamberg CJ, Roth CP, Solomon DH, et al Quality of care is associated with survival in vulnerable older patients. Annals of Internal Medicine. 2005;143(4):274–281. 16103471 10.7326/0003-4819-143-4-200508160-00008

[13] Rossi MC, Lucisano G, Comaschi M, Coscelli C, Cucinotta D, Di Blasi P, et al Quality of diabetes care predicts the development of cardiovascular events: results of the AMD-QUASAR study. Diabetes Care. 2011;34(2):347–352. 10.2337/dc10-1709 21270192 10.2337/dc10-1709 PMC3024347

[14] Han W, Sharman R, Heider A, Maloney N, Yang M, Singh R. Impact of electronic diabetes registry 'Meaningful Use' on quality of care and hospital utilization. J Am Med Inform Assoc. 2016;23(2):242–7 10.1093/jamia/ocv040 26133895 10.1093/jamia/ocv040 PMC11740534

[15] Cederholm J, Zethelius B, Nilsson PM, Eeg-Olofsson K, Eliasson B, Gudbjörnsdottir S; Swedish National Diabetes Register. Effect of tight control of HbA1c and blood pressure on cardiovascular diseases in type 2 diabetes: An observational study from the Swedish National Diabetes Register (NDR). Diabetes Res Clin Pract. 2009 10;86(1):74–81. 10.1016/j.diabres.2009.07.003 19679369 10.1016/j.diabres.2009.07.003

[16] Stratton IM, Adler AI, Neil HA, Matthews DR, Manley SE, Cull CA, et al Association of glycaemia with macrovascular and microvascular complications of type 2 diabetes (UKPDS 35): prospective observational study. BMJ. 2000;321(7258):405–412. 10938048 10.1136/bmj.321.7258.405 PMC27454

[17] Rosen B, Porath A, Pawlson LG, Chassin MR, Benbassat J: Adherence to standards of care by health maintenance organizations in Israel and the USA. Int J Qual Health Care 2011, 23:15–25. 10.1093/intqhc/mzq065 21084320 10.1093/intqhc/mzq065

[18] Shalev V, Chodick G, Goren I, Silber H, Kokia E, Heymann AD. The use of an automated patient registry to manage and monitor cardiovascular conditions and related outcomes in a large health organization. Int J Cardiol. 2011;152(3):345–9. 10.1016/j.ijcard.2010.08.002 20826019 10.1016/j.ijcard.2010.08.002

[19] Chodick G, Heymann AD, Rosenmann L, Green MS, Flash S, Porath A, et al Diabetes and risk of incident cancer: a large population-based cohort study in Israel. Cancer Causes Control (2010) 21(6):879–887. 10.1007/s10552-010-9515-8 20148361 10.1007/s10552-010-9515-8

[20] Friedman NL, Kokia E, Shemer J. Health Value Added (HVA): linking strategy, performance, and measurement in healthcare organizations. Isr Med Assoc J. 2003;5(1):3–8. 12592948

[21] Heymann AD, Chodick G, Halkin H, Karasik A, Shalev V, Shemer J, et al The implementation of managed care for diabetes using medical informatics in a large Preferred Provider Organization. Diabetes Res Clin Pract 2006;71(3):290–8. 10.1016/j.diabres.2005.07.002 16112245 10.1016/j.diabres.2005.07.002

[22] Chodick G, Heymann A, Shalev V, Kookia E. The epidemiology of diabetes in a large Israeli HMO. Eur J Epidemiol 2003;18(12):1143–6. 14758871 10.1023/b:ejep.0000006635.36802.c8

[23] Association AD. Standards of medical care for patients with diabetes mellitus. Clin Diabetes. 2002;20(1):24–33.

[24] Jha AK, Perlin JB, Kizer KW, Dudley RA. Effect of the transformation of the veterans affairs health care system on the quality of care. N Engl J Med 2003, 348(22):2218–2227. 10.1056/NEJMsa021899 12773650 10.1056/NEJMsa021899

[25] Kerr EA, Gerzoff RB, Krein SL, Selby JV, Piette JD, Curb JD, et al Diabetes care quality in the veterans affairs health care system and commercial managed care: the TRIAD study. Arch Inter Med 2004, 141:272–281. 10.7326/0003-4819-141-4-200408170-00007 15313743

[26] Larsson S, Lawyer P, Garellick G, Lindahl B, Lundstrom M. Use of 13 disease registries in 5 countries demonstrates the potential to use outcome data to improve health care’s value. Health Aff (Millwood). 2012;31(1):220–7. 22155485 10.1377/hlthaff.2011.0762

[27] Emilsson L, Lindahl B, Koster M, Lambe M, Ludvigsson JF. Review of 103 Swedish Healthcare Quality Registries. J Intern Med. 2015;277(1):94–136. 10.1111/joim.12303 25174800 10.1111/joim.12303

[28] Lind M, Garcia-Rodriguez L, Booth G, Cea-Soriano L, Shah B, Ekeroth G, et al Mortality trends in patients with and without diabetes in Ontario, Canada and the UK from 1996 to 2009: a population-based study. Diabetologia. 2013;56(12):2601–8. 10.1007/s00125-013-3063-1 24114114 10.1007/s00125-013-3063-1

[29] Færch K, Carstensen B, Almdal TP, Jørgensen ME. Improved survival among patients with complicated type 2 diabetes in Denmark: a prospective study (2002–2010). J Clin Endocrinol Metab.2014;99(4):E642–6 10.1210/jc.2013-3210 24483155 10.1210/jc.2013-3210

